# Expansion Microscopy‐based imaging for visualization of mitochondria in *Drosophila* ovarian germline stem cells

**DOI:** 10.1002/2211-5463.13506

**Published:** 2022-11-17

**Authors:** Chi‐Hung Lin, Tzu‐Yang Lin, Shao‐Chun Hsu, Hwei‐Jan Hsu

**Affiliations:** ^1^ Molecular and Cell Biology, Taiwan International Graduate Program Academia Sinica Taipei Taiwan; ^2^ Graduate Institute of Life Science National Defense Medical Center Taipei Taiwan; ^3^ Institute of Cellular and Organismic Biology Academia Sinica Taipei Taiwan; ^4^ Branch Office of Research and Development National Taiwan University College of Medicine Taipei Taiwan

**Keywords:** *Drosophila*, ExM, expansion microscopy, GSC, mitochondria, ovary

## Abstract

Recent studies have shown that mitochondrial morphology can modulate organelle function and greatly affect stem cell behavior, thus affecting tissue homeostasis. As such, we previously showed that the accumulation of fragmented mitochondria in aged *Drosophila* ovarian germline stem cells (GSCs) contributes to age‐dependent GSC loss. However, standard immunofluorescence methods to examine mitochondrial morphology yield images with insufficient resolution for rigorous analysis, while 3‐dimensional electron microscopy examination of mitochondrial morphology is labor intensive and allows only limited sampling of mitochondria. To overcome these issues, we utilized the expansion microscopy technique to expand GSC samples by 4‐fold in combination with mitochondrial immunofluorescence labeling. Here, we present a simple, inexpensive method for nanoscale optical imaging of mitochondria in the germline. This protocol may be beneficial for studies that require visualization of mitochondria or other fine subcellular structures in the *Drosophila* ovary.

## Graphical abstract

We have established a 4X expansion microscopy method to expand Drosophila ovaries. After immunofluorescence labeling of mitochondria (mito, green) followed by gelation, digestion and expansion steps, the resolution of mitochondria is largely improved in germ cells, including germline stem cells (GSCs).

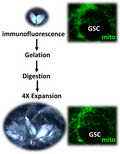

**Abbreviations**
4HT: 4‐Hydroxy‐TEMPO
AcX: Acryloyl X‐SE
APS: ammonium persulfate
BIS: *N*,*N*′‐methylenebisacrylamide
BSA: bovine serum albumin
DMSO: anhydrous dimethyl sulfoxide
ExM: expansion microscopy
GIM: Grace's insect medium
GSC: germline stem cell
Lam: lamin
TEMED: *N*,*N*,*N*′,*N*′‐tetramethylethylenediamine
EDTA: ethylenediaminetetraacetic acid

Stem cells play a critical role in maintaining tissue homeostasis, at least in part due to their capacity to differentiate and replenish lost cells in the tissue. Recent studies have also shown that stem cell maintenance and differentiation processes are highly dependent on mitochondrial ATP production and dynamics (fission, one mitochondrion becomes two mitochondria; fusion, two mitochondria become one) [1, 2]. For example, disruption of mitochondrial fusion in murine neural stem cells impairs their self‐renewal capability [1]. In addition, our group reported that shifting mitochondrial dynamics toward fission in aged *Drosophila* ovarian germline stem cells (GSCs) promotes GSC loss [3]. While previous studies have mostly utilized immunofluorescence to visualize mitochondria, this approach yields images with relatively low resolution.

Expansion Microscopy (ExM) is an imaging protocol to ‘make the specimen bigger,’ that was first developed by the Boyden group [4]. At present, ExM protocols for 4X expansion are well established, and the approach has been successfully used for expanding cultured cells, tissues and organs [5, 6, 7]. This method has been especially useful for visualizing subcellular structures. For instance, a previous study used ExM with ~ 4X expansion factor to investigate protein assembly in the synaptonemal complex [8]. This complex is critical for chromosome segregation during meiosis [9] in the *Drosophila* germarium, where female GSCs reside (Fig. 1A). Nevertheless, no detailed procedure for performing ExM on germarial tissues has yet been published.

**Fig. 1:**
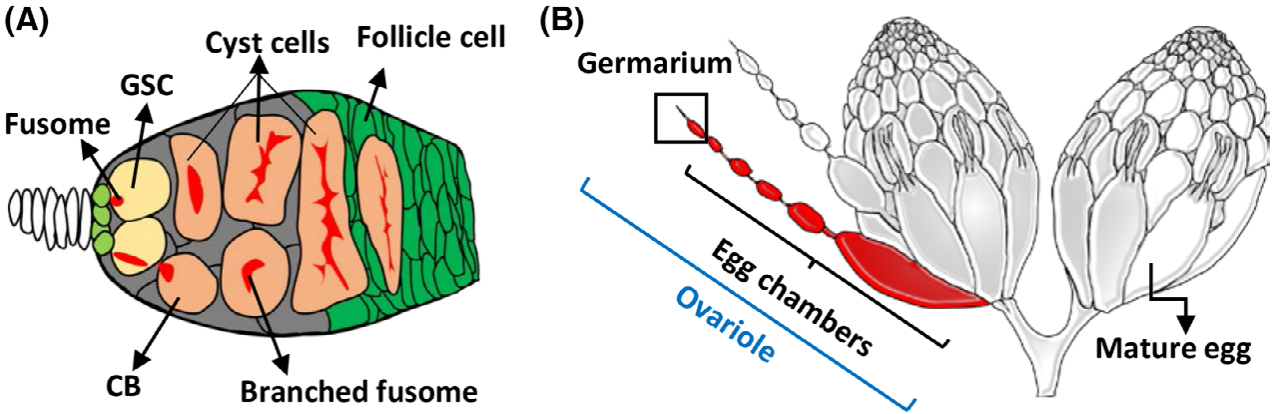
The *Drosophila* germarium and ovary. The *Drosophila* germarium (A) is the anterior‐most structure of the ovary (B). Two or three GSCs reside in the anterior tip of the germarium. Each GSC carries a membrane‐enriched organelle, called a fusome. The immediate GSC daughter cell (cystoblast, CB) undergoes four rounds of incomplete mitosis to become a 2‐, 4‐, 8‐ and 16‐cell cyst; germ cells within the cyst are interconnected by a single branching fusome. The entire cyst is surrounded by follicle cells and buds off from the germarium to form a newly formed egg chamber. The egg chamber eventually develops into a mature egg. The germarium and its associated string of differently staged egg chambers are called an ovariole; 16–20 ovarioles comprise an ovary.

The *Drosophila* female GSC is an excellent stem cell model, as it has well‐characterized cell biology and many available genetic tools [10]. GSCs are located at the anterior tip of the germarium, and the cells can be recognized by their fusome (labeled by anti‐Hts antibody), which is adjacent to the GSC‐supporting cap cells [labeled by anti‐Lamin (Lam) C antibody] [11, 12]. The immediate GSC progeny undergoes four rounds of incomplete division to become a 16‐cell cyst [13]; each germ cell within the cyst is interconnected by a branching fusome. After its formation, the 16‐cell cyst is surrounded by follicle cells and buds off from the germarium to become an egg chamber, which passes through 14 different developmental stages and finally develops into a mature egg. The entire structure of the germarium and string of egg chambers is called the ovariole. About 16–20 ovarioles can be found in a typical ovary (Fig. 1B) [14]. To track mitochondrial shape during the process of egg production from GSCs, we modified the available 4X ExM protocol (http://expansionmicroscopy.org/) to establish the step‐by‐step protocol we report here. Because we were able to successfully expand the entire ovary, this protocol may be used for studies of fine structure in GSCs and other ovarian cells.

## Materials

### Sample preparation

#### *Drosophila* strains and culture

1. Bloomington Drosophila Stock Center standard cornmeal medium.
2. Active yeast power (ML 262607001002; Yung Cheng Industries Ltd, Taipei, Taiwan).

#### Immunofluorescence

1. Grace's Insect Medium (GIM; #11605‐904; Gibco/Thermo Fisher, Billings, MT, USA).
2. 16% Paraformaldehyde (#50‐00‐0; Alfa Aesar/Thermo Fisher, Tewksbury, MA, USA).
3. Triton X‐100 (#9036‐19‐5; Sigma‐Aldrich, Burlington, MA, USA).
4. 10× PBS (#UR‐PBS001‐5L; UniRegion Bio‐Tech, New Taipei City, Taiwan).
5. Blocking Buffer (#W‐3400; GoalBio, Taipei, Taiwan).
6. Bovine serum albumin (BSA; #9048‐46‐8; Bioshop, Burlington, Canada).
7. Glycerol (#56‐81‐5; Sigma‐Aldrich).
8. *n*‐propyl gallate (121‐79‐9; Sigma‐Aldrich).
9. Mouse monoclonal anti‐ATP5α antibody [15H4C4] (#ab14748; Abcam, Biomedical Campus, Cambridge, UK).
10. Mouse monoclonal anti‐Lam C antibody (#LC28.26; Developmental Studies Hybridoma Bank, Iowa City, IA, USA).
11. Mouse monoclonal anti‐Hts antibody (#1B1; Developmental Studies Hybridoma Bank).
12. Goat anti‐mouse IgG (H + L) cross‐adsorbed secondary antibody, Biotin‐XX (#B2763; Thermo Fisher Scientific, Waltham, MA, USA).
13. Streptavidin, Alexa Fluor™ 488 conjugate (#S11223; Thermo Fisher Scientific).
14. Streptavidin, Alexa Fluor™ 568 conjugate (#S11226; Thermo Fisher Scientific).
15. DAPI (#28718‐90‐3; Sigma‐Aldrich).

### Gelation, digestion and expansion

1. Acryloyl X‐SE (AcX; #A20770; Life Technologies, Carlsbad, CA, USA).
2. Anhydrous dimethyl sulfoxide (DMSO; #67‐68‐5; Sigma‐Aldrich).
3. Sodium acrylate (#408220; Sigma‐Aldrichs).
4. Acrylamide (#49099; Sigma‐Aldrich).
5. *N*,*N*′‐Methylenebisacrylamide (BIS; #M7279; Sigma‐Aldrich).
6. Ammonium Persulfate (APS; #A3678; Sigma‐Aldrich).
7. *N*,*N*,*N*′,*N*′‐Tetramethylethylenediamine (TEMED; #T7024; Sigma‐Aldrich).
8. 4‐Hydroxy‐TEMPO (4HT; #176141; Sigma‐Aldrich).
9. Proteinase K (#P8107S; New England Biolabs, Ipswich, MA, USA).
10. Sodium chloride (#7647‐14‐5; MERCK, Kenilworth, NJ, USA).
11. Ethylenediaminetetraacetic acid (EDTA; #60‐00‐4; Sigma‐Aldrich).
12. Tris (Base; #77‐86‐1; J.T.Baker, Phillipsburg, NJ, USA).
13. Poly‐L‐lysine solution (#25988‐63‐0; Sigma‐Aldrich).

### Imaging

#### Acquisition

1. Confocal microscopy Zeiss 900 with Airyscan 2
2. Objective LD LCI Plan‐Apochromat 63×/1.2NA Imm Corr DIC M27 (#420882‐9870‐799; Zeiss, Oberkochen, Germany)

#### Image processing and analysis

Software:

1. fiji (NIH, Bethesda, MD, USA)
2. Zen 3.3 (blue edition, Zeiss)
3. Microsoft Excel

### Miscellaneous materials

**Table:** 

Fly handling
Paintbrush
Zeiss stereo microscope (Zeiss Stemi 305)
Ovaries dissection, immunostaining, gelation and mounting
1.5‐mL centrifuge tubes
Forceps
25G gauge needle and syringe
35‐mm Petri dishes
Nutator
Pasteur pipette
Aluminum foil
Kimwipes
Heating plate
Ice bath
Clay
Microscope slides
24 × 50 mm coverslips
22 × 22 mm coverslips
Paper hole reinforcing ring

## Methods

### Sample preparation

#### *Drosophila* strains and culture

Maintain the *yw* strain on Bloomington Drosophila Stock Center standard cornmeal medium at 22–25 °C. Culture 10 newly eclosed female flies with five males on standard medium with a paste of wet yeast for 7 days; change food daily.

#### Immunofluorescence

##### Solutions prepared for this step

1. 30% w/v BSA stock solution: dissolve 15 g BSA in 50 mL ddH_2_O in a 50‐mL conical tube. Store at 4 °C.
2. 3% BSA stock solution: Mix 5 mL 30% BSA with 45 mL ddH_2_O in a 50‐mL conical tube. Store at 4 °C.
3. 10% Triton X‐100: Mix 5 mL 100% Triton X‐100 with 45 mL ddH_2_O in a 50‐mL conical tube. Store at room temperature (RT).
4. 1× PBS: Mix 5 mL 10× PBS with 45 mL ddH_2_O in a 50‐mL conical tube. Store at RT.
5. 1× PBST: Mix 5 mL 10× PBS and 500 μL 10% Triton X‐100 with 44.5 mL ddH_2_O in a 50‐mL conical tube. Store at RT.
6. 16% paraformaldehyde: Aliquot 16% paraformaldehyde as 1‐mL stocks. Store at −20 °C.
7. 5.3% paraformaldehyde: Mix 500 μL 16% paraformaldehyde with 1 mL GIM for immediate use.
8. Mounting solution: Add 1 g *n*‐propyl gallate to 7.5 mL PBS and vortex in a 50‐mL conical tube. After the *n*‐propyl gallate is completely dissolved, add 42.5 mL 100% glycerol. Wrap the tube with aluminum foil and rotate overnight (O/N) at 4 °C.

##### Experimental procedure

1. Coat centrifuge tubes for collecting dissected ovaries with 3% BSA for 5 min to prevent the tissues from sticking to the tube wall.
2. Dissect 10 pairs of ovaries in prewarmed Grace's Insect Medium (GIM) on a 35‐mm plastic plate using a pair of sharp forceps [15]. Transfer the dissected tissues to coated 1.5‐mL centrifuge tubes using a Pasteur pipette. Fix the ovaries with 5.3% paraformaldehyde in GIM at RT for 13 min on a nutator with gentle agitation (see Note 1).
3. Wash the fixed ovaries three times with PBST (0.1% Triton X‐100 in 1× PBS) for 30 min each wash (see Note 2). After washing, tease apart ovaries on the plastic plate using a 25G gauge needle and syringe. Then, transfer the ovaries back to the centrifuge tube and incubate with blocking buffer O/N.
4. Incubate ovaries with mouse anti‐ATP5α antibody (1 : 500 dilution in blocking buffer) at 4 °C O/N. After three washes with PBST, incubate ovaries with anti‐mouse IgG (H + L) cross‐adsorbed secondary antibody, Biotin‐XX (1 : 200 dilution in blocking buffer) at 4 °C O/N (see Note 3). After three washes with PBST, incubate ovaries with Streptavidin, Alexa Fluor™ 488 conjugate (1 : 200 dilution in 1× PBS) at 4 °C O/N.
5. After three washes with PBST, incubate ovaries with mouse anti‐LamC antibody (1 : 50 dilution in blocking buffer) at 4 °C O/N. Then, incubate the samples with mouse anti‐Hts antibody (1 : 50 dilution in blocking buffer) at 4 °C O/N. After three washes with PBST, incubate ovaries with anti‐mouse IgG (H + L) cross‐adsorbed secondary antibody, Biotin‐XX (1 : 200 dilution in blocking buffer) at 4 °C O/N. After three washes with PBST, incubate the samples with Streptavidin, Alexa Fluor™ 568 conjugate (1 : 200 dilution in 1× PBS) at 4 °C O/N.
6. Without washing, directly stain ovaries with 0.5 mg·mL^−1^ DAPI in 1× PBS for 20 min at RT. After removing DAPI solution, pipet ~ 200 μL of mounting solution (80% glycerol containing 20 mg·mL^−1^ *n*‐propyl gallate) into the centrifuge tube. Ovaries with mounting solution may be immediately used for the next step or kept at −20 °C for up to 2 weeks (see Note 4).

### Gelation, digestion and expansion

#### Solutions prepared for this step

1. AcX stock solution (10 mg·mL^−1^): dissolve 5 mg AcX in 500 μL anhydrous dimethyl sulfoxide (DMSO). Divide the solution into 10 μL aliquots and store at −20°C for up to 2 months (see Note 5).
2. Acrylamide stock solution: Dissolve 50 g acrylamide in 100 mL ddH_2_O with vortexing but not heating. Store at 4 °C for up to 6 months.
3. *N*,*N*′‐methylenebisacrylamide stock solution: Dissolve 2 g *N*,*N*′‐methylenebisacrylamide in 100 mL ddH_2_O with vortexing but not heating. Store at 4 °C for up to 6 months.
4. 5 m Sodium chloride stock solution: Dissolve 29.2 g sodium chloride in 100 mL ddH_2_O.
5. 4HT stock solution: Dissolve 0.5 g 4HT in 100 mL ddH_2_O. The solution can be divided into 10 mL aliquots and stored at −20 °C for up to 2 weeks.
6. TEMED stock solution: Dissolve 1 g TEMED in 10 mL ddH_2_O. Divide the solution into 20 μL aliquots. Store at −20 °C for up to 2 weeks.
7. APS stock solution: Dissolve 1 g APS in 10 mL ddH_2_O. Divide into 20 μL aliquots, and store at −20 °C for up to 2 weeks.
8. 0.5 m EDTA (pH 8.0): Dissolve 186.1 g EDTA into 800 mL ddH_2_O. Add 1 m NaOH to adjust the pH to 8.0. Then, add ddH_2_O to 1 L.
9. 1 m Tris (PH 8.0): Dissolve 209.24 g Tris in 750 mL ddH_2_O. Adjust the pH to 8.0 using 1 m HCl. Then, add ddH_2_O to 1 L.
10. 10% Triton X‐100: Add 5 mL 100% Triton X‐100 to 45 mL ddH_2_O and mix well.
11. Sodium acrylate freshly made solution: Dissolve 38 g sodium acrylate in 100 mL ddH_2_O (see Note 6).
12. Monomer solution [called Stock X]

**Table:** 

Sodium acrylate solution	2.25 mL
Acrylamide stock solution	0.5 mL
*N*,*N*′‐methylenebisacrylamide stock solution	0.75 mL
5 m sodium chloride	4 mL
10× PBS	1 mL
ddH_2_O	0.9 mL
Total	9.4 mL

The Stock X solution may be divided into aliquots of 950 μL each and stored at −20 °C for up to 6 months.

1. Gelling solution

**Table:** 

Stock X	188 μL
TEMED stock solution	4 μL
4TH stock solution	4 μL
APS stock solution	4 μL
Total	200 μL

The gelling solution is made by adding Stock X, TEMED, 4HT and APS (in order) with vortexing. The solution should be freshly prepared and kept on the ice to prevent premature polymerization of the gel. The amount of gelling solution can be scaled up or down according to the need. The APS should be added last to avoid polymerization during the preparation.

1. Digestion buffer

**Table:** 

10% Triton X‐100	5 mL
0.5 m EDTA (PH8)	1 mL
1 m Tris (PH8)	25 mL
5 m sodium chloride	4 mL
ddH_2_O	Add up to 500 mL
Proteinase K	1 : 100 dilution

The digestion buffer without Proteinase K can be stored in aliquots at −20 °C for a few months. Proteinase K can be stored at 4 °C and should be added to digestion buffer immediately before the digestion step.

#### Experimental procedure

##### Gelation

**Fig. 2:**
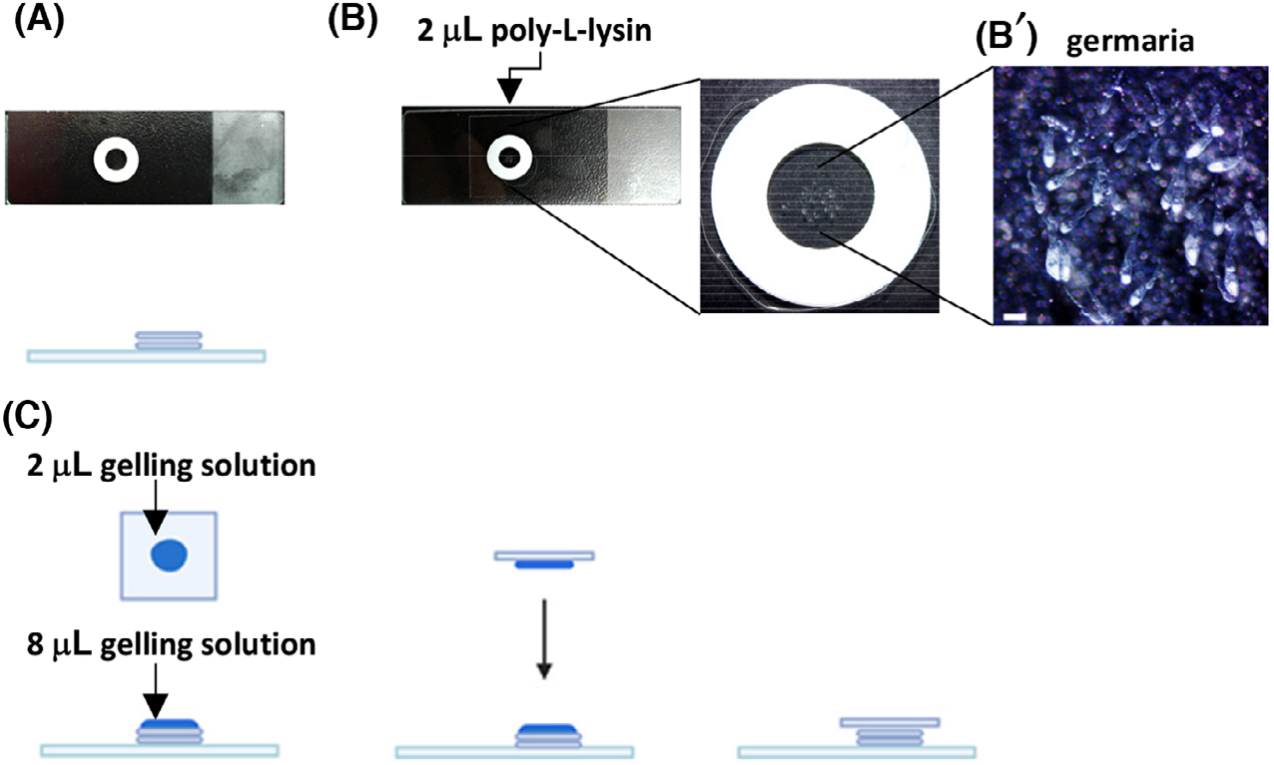
Key steps for the gelation process. (A) Stack two reinforcing rings on the glass slide. (B) Add 2 μL poly‐l‐lysine to the center of the chamber made by the reinforcing rings, and let it dry. (B') Enlarged view of the gelation chamber. After removal of the egg chambers, germaria are arranged in the center of the chamber. Scale bar is 200 μm. (C) Add 8 μL gelling solution with APS to the germaria in the chamber, and add 2 μL gelling solution to the coverslip. Hold the coverslip with coated side down and gently lower it to cover the sample.

1. Wash ovaries with PBST three times at RT; each wash should be 30 min.
2. Post‐fix ovaries to crosslink fluorescence labels to the gel. For post‐fixation, incubate samples in 1 mL 1× PBS containing 10 μL AcX stock solution (final AcX concentration is 0.1 mg·mL^−1^) for O/N at 4 °C.
3. Wash ovaries twice with 1× PBS; each wash should be 15 min.
4. Add gelling solution (without APS), and keep ovaries in the dark on ice for 30 min until all the tissues sink to the bottom of the centrifuge tube.
5. During the 30‐min incubation, prepare the gelation chamber by stacking two paper hole reinforcing rings on a glass slide (Fig. 2A). Then, add 2 μL poly‐l‐lysine solution onto the center of the reinforcing rings on the slide (Fig. 2B). Dry the slides at 55 °C using a heating plate.
6. After incubation, transfer 2 or 3 ovaries with gelling solution (without APS) onto the gelation chamber using a Pasteur pipette. Remove egg chambers using a pair of 25G needles with syringes. The remaining germaria can be pushed into the center of the gelation chamber (Fig. 2B'; see Note 7). At this position, the coated poly‐l‐lysine should adhere to the germaria.
7. Remove the gelling solution (without APS) from the gelation chamber using a 20‐μL pipetman, and add approximately 8 μL gelling solution with APS to the gelation chamber (see Notes 8 and 9).
8. Add 2 μL gelling solution (with APS) to one side of the coverslip (22 × 22 mm). Then, flip the coverslip over and hold it horizontally to gently cover the gelation chamber (Fig. 2C;see Note 10).
9. Place the gelation chamber at RT for approximately 4 h to allow polymerization.
10. For pre‐expansion imaging of germaria during polymerization, use a Zeiss LSM 900 Confocal Scanning Laser Microscopy equipped with a 63×/1.2 NA Plan‐Apochromat oil immersion objective in Airyscan mode, or equivalent. Pinhole size may be set to 1 AU, and multichannel z‐stack with optimal z‐interval images may be acquired using the 488 nm laser (ATP5α‐labeled mitochondria), the 568 nm laser (Hts‐ and LamC‐labeled fusomes and cap cell nuclear envelopes, respectively) and the 405 nm laser (DAPI‐labeled DNA). In our set‐up, the 488 nm was used at 0.4% power.

##### Digestion

1. Insert a razor blade into the space between the coverslip and reinforcing rings to pry up the coverslip.
2. Remove the reinforcing rings, and trim the blank gel region around the germaria using a razor blade.
3. Add 2 mL digestion buffer in a 35‐mm Petri dish, and then add 20 μL proteinase K before mixing well.
4. Wet the gel with digestion buffer, and use a paintbrush to separate the gel from the slide. Using the paintbrush, transfer the gel to the 35‐mm Petri dish containing digestion buffer. Rotate the samples O/N at RT in the dark.
5. After removing the digestion buffer, add 20 mL 1× PBS to the 35‐mm Petri dish. Store the gel at 4 °C in the dark for up to 3 days.

##### Expansion

1. Remove the 1× PBS, and replace it with ddH_2_O. Then, leave the 35‐mm Petri dish with the gel on a rotator with gentle rotation for 20 min at RT. Repeat 3–4 times and incubate for 20 min each time at RT. The expanded gel can be kept in the dark at 4 °C for up to 2 days.

### Acquisition

#### Post‐expansion imaging

**Fig. 3:**
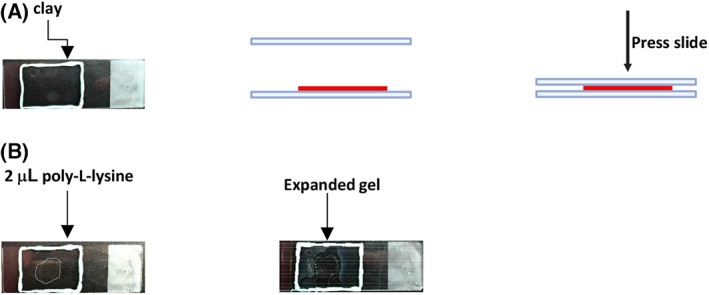
Steps for mounting expanded gel on the slide. (A) Use clay to make a rectangular edge on the glass slide. Use another slide to flatten the clay. (B) Add 2 μL poly‐l‐lysine on the center, and allow it to dry. Transfer the expanded gel with a paintbrush, and fill the cavity with ddH_2_O. Cover the sample with a long coverslip.

1. Slide preparation: Use clay to make a rectangular edge on the glass slide. Then, use another glass slide to flatten the clay edge (Fig. 3A). Add 2 μL poly‐l‐lysine on the center of the slide, and dry the slide at 55 °C on a heating plate (Fig. 3B).
2. Transfer the expanded gel from the Petri dish to the center of the glass slide and fill the clay rectangle with water. Cover the expanded gel with a long coverslip (24 × 50 mm) and remove excess water. Wrap the slide with wet Kimwipes for storage at 4 °C for up to 3 days.
3. Acquire z‐stack images of germaria using the Zeiss LSM 900 confocal laser scanning microscope equipped with a 63×/1.2 NA Plan‐Apochromat oil immersion objective in Airyscan mode, or equivalent. Pinhole size may be set to 1 AU, and multichannel z‐stack with optimal z‐interval images should be taken using the 488 nm laser (ATP5α), the 568 nm laser (Hts and LamC) and the 405 nm laser (DNA). In our set‐up, the 488 nm laser was used at 2% power.

#### Image processing and analysis

Images of germaria before and after expansion were taken using the Zeiss confocal microscope with Airyscan module. Deconvolution was performed using Zeiss zen 3.3 (blue version). Images were then opened in fiji, and a threshold was set at two times the length of the smallest mitochondrion (15 pixels or below was considered noise). Image analyses were performed according to a previous study [16]. In brief, lines of the same length (550 pixels) were drawn through a GSC before and after expansion. The areas without signal at both ends and the center of the line were used for measuring noise. Plot profile was selected in fiji for each line, which generates plots of pixel intensity along the line. The data for each line in the GSC before and after expansion were processed into a graph using Excel. A signal‐to‐noise ratio was calculated for each plot profile; noise was defined based on the average pixel intensity of the first two, middle two and the last two pixels of the line. Mitochondrial signal was defined as one standard deviation above the mean intensity of the plot profile. The full width at half maximum height of the tallest signal peak in each plot profile was measured in pixels. The expansion factor was then calculated by dividing the thickness (z‐stack) of each germanium before and after expansion.

## Results and discussion

**Fig. 4:**
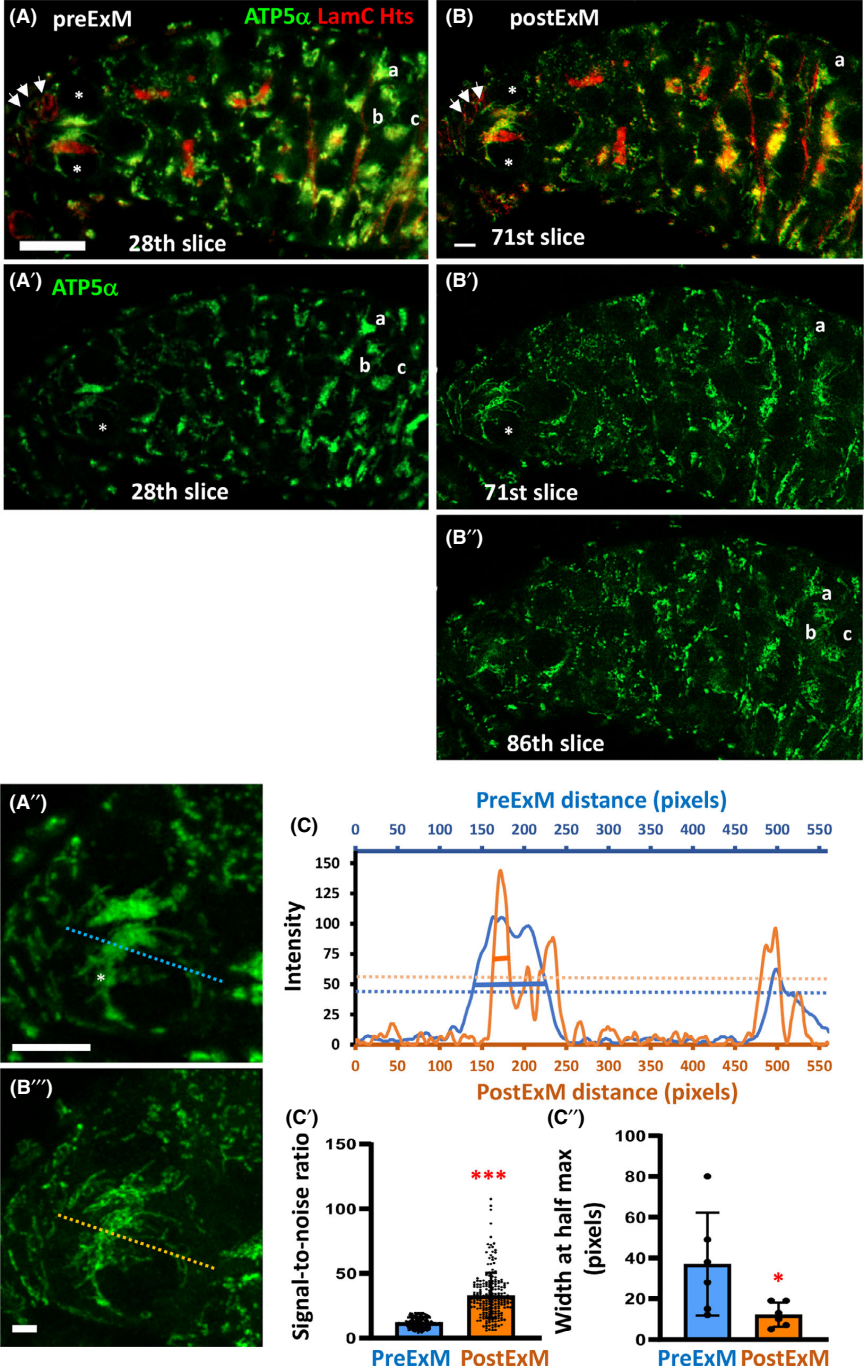
Expansion microscopy improves visualization of mitochondria in the germline. Representative images of a one‐week‐old germarium before expansion (preExM, the 28th slice) (A) and after expansion (postExM, the 71th slice) (B). ATP5α labels mitochondria (green); Hts labels fusomes (red); LamC labels the nuclear envelope of GSC niche cells (red; indicated by arrows); DAPI labels DNA (not shown). GSCs are marked by asterisks. A' and B' show only the ATP5α channel of A and B. B'' shows the ATP5α channel of the 86th slice. Cells labeled with a, b and c are the same posterior germ cells as shown in A (preExM) and were present in the 86th slice of the expanded germarium (B''). A" and B''' are enlarged views of GSCs shown in only the green channel. (C) The representative plot profile of pixel intensity through mitochondria of the same GSCs shown in A" and B"' before (blue) and after (orange) expansion. Dashed lines show the thresholds to define signal versus noise. Solid lines show the width of the highest peak at half maximum value for preExM and postExM lines. (C') The signal‐to‐noise ratios were measured from plot profiles generated from six lines passing through the GSC before and after expansion; as shown in A' and B'. (C'') The full width of the tallest peak at half maximum value in the plot profiles generated from six lines passing through the GSC before and after expansion; as shown in A' and B'. Error bars in C' and C'' are standard deviation. Statistical differences in C' and C'' were analyzed by two‐tailed Student's *t*‐test. *, *P* < 0.05; ***, *P* < 0.001. A and B are images of the same germarium. Scale bars in A and B are 10 μm, and in A' and B' are 5 μm.

To determine whether expansion affected germarial morphology and subcellular structures (i.e., fusomes and mitochondria), we arranged germaria in a specific pattern for the gelation step (see Fig. 2B). After gelation, we captured images of germaria that were arranged horizontally at the bottom of the gel (Fig. 4A shows the 28th slice of the 38‐slice z‐stack). Then, we took images of the same germaria after expansion (Fig. 4B shows the 71st slice of the 133‐slice z‐stack). After expansion, the mitochondrial fluorescent signal intensity is decreased, but resolution is increased for both anterior and posterior germ cells (Fig. 4A',B'). This effect is presumably due to the decrease in density of fluorescent molecules within the expanded tissue. In addition, after expansion, the posterior germ cells (marked by a, b, c) in the 28th slice of the unexpanded germarium (see Fig. 4A,A') were not present in the same slice as the marked GSCs (see Fig. 4B,B'). Instead, these cells were present in the 86th slice of the z‐stack (Fig. 4B"). To calculate the expansion factor, we compared the thickness of the germarium after expansion (obtained by z‐stack set by the confocal) to that of the germarium before expansion. We found that our protocol routinely expanded the germarium by a factor of 3.88 ± 0.4. Although we did see a slight change in germarial shape after expansion (Fig. 4A,B), mitochondria could be seen more easily without obvious distortion after the expansion (Fig. 4A'',B'''). To confirm that mitochondria maintained their shapes, we measured the micro‐expansion of the GSC by applying the plot profile tool on lines of the same length (550 pixels) drawn through the GSC before and after expansion (Fig. 4A',B'). The intensity of each pixel along the line of preExM GSCs was scaled in X–Y space (with fiji) to match the pixel dimensions of the postExM GSC (Fig. 4C). We converted pixels along the line drawn in preExM and postExM images into microns and divided the length of the postExM plot profile line in microns by the length of the preExM plot profile line to determine the expansion factor (3.2 ± 0.2). In summary, our procedure reliably yielded macro‐expansion factors of 3.8 and micro‐expansion factors of 3.2.

To demonstrate the enhanced resolution of mitochondrial structure after expansion, we measured the signal‐to‐noise ratio (Fig. 4C'). Positive mitochondria signals were defined as one standard deviation above the mean (shown as a dashed line in Fig. 4C). Noise was calculated from the area without signal (two pixels from both ends, and at the center of the line). The average signal‐to‐noise ratio was increased by about 3‐fold upon expansion (Fig. 4C', 5 K); this increase in signal‐to‐noise ratio mostly reflected a decrease in noise. Furthermore, the full width at half maximum of the highest peak in each plot profile was calculated (Fig. 4C"; See solid line in Fig. 4C). After expansion, the peak width at half maximum was reduced by about 4‐fold, indicating sharper and more defined signal peaks. Overall, our expansion procedure greatly increased the resolution of mitochondrial structure. However, we noticed that even with 3.2‐fold expansion of the GSC, mitochondria were still packed very tightly (see Fig. 4B), suggesting that even greater expansion of GSCs may be required for highly detailed studies of mitochondrial structure.

## Tips &Tricks

Note 1 During fixation, washing and antibody incubation, all reagents except mounting solution can be used at 1 mL; samples may be left on a nutator with gentle rotation (~ 12 r.p.m.).

Note 2 The washing time can vary, but each should be at least 30 min.

Note 3 Ovaries incubated with streptavidin‐conjugated fluorophore or DAPI may be protected from light with aluminum foil.

Note 4 We did not test expansion of samples that were stored at −20 °C for longer than 2 weeks.

Note 5 The AcX stock is highly sensitive to temperature; once an aliquot is removed from −20 °C, the unused AcX should be discarded.

Note 6 The solution may appear light yellow, but the color does not appear to affect expansion.

Note 7 Germaria located in the middle or top of the gel will be beyond the working distance of a high‐magnification objective.

Note 8 The gelling solution should be freshly prepared and kept on the ice. Avoid excessive warming of the gelling solution to prevent premature polymerization of the gel.

Note 9 When using a pipetman to remove the gelling solution (without APS) from the gelation chamber, take care to avoid picking up the samples. Add gelling solution (with APS) to the gelation chamber slowly in order to prevent the samples from floating up.

Note 10 Slowly lower the horizontal coverslip until the gelling solution on the coverslip fuses with the gelling solution on the gelation chamber. This step is critical to eliminate air pockets in the gelation chamber.

## Conflict of interest

The authors declare no conflict of interest.

## Author contributions

C‐HL, T‐YL, S‐CH and H‐JH designed and interpreted the experiments and wrote the paper. C‐HL performed the experiments; S‐CH assisted with image analysis.

## Data Availability

The source data that support the findings of this study are available from the corresponding author upon reasonable request.
